# Age and Body Mass Index: the most important factors of urinary and erectile function recovery after robotic assisted radical prostatectomy

**DOI:** 10.1590/S1677-5538.IBJU.2019.04.01

**Published:** 2019-09-02

**Authors:** Luciano A. Favorito

**Affiliations:** MD, PhD. Professor Associado da Unidade de Pesquisa Urogenital da Universidade do Estado de Rio de Janeiro Brasil; Urologista do Hospital da Lagoa Federal, Rio de Janeiro. Editor Associado da International Braz J Urol Brasil

The July-August 2019 issue of the International Brazilian Journal of Urology presents original contributions with a lot of interesting papers in different fields: Infertility, Bladder augmentation, Bladder Cancer, PCNL, Prostate Cancer, Renal Cell Carcinoma, Partial nephrectomy, Renal stones, Nocturnal Enuresis, Basic Research, Laparoscopic Surgery, Penile Cancer, Stress Urinary Incontinence and Adrenalectomy. The papers come from many different countries such as Italy, Brazil, USA, UK, Turkey, China, France, Iran, Republic of Korea, Argentina, India and Spain, and as usual the editor´s comment highlights some papers. We decided to comment the paper about a very interesting topic: Robotic-Assisted Radical Prostatectomy (RARP).

Doctor Neumaier and collegues from the FMUSP, Brazil performed on page 703 (1) an interesting study about the factors involved in urinary continence and sexual potency recovery after robotic-assisted radical prostatectomy (RARP). They studied 104 patients operated by two surgeons between 2008 and 2015, with a minimum 12 months follow-up. The patient features (age, body mass index, PSA, date of surgery and sexual function), tumor features (tumor stage, Gleason and surgical margins) and follow-up data (time to reach urinary continence and sexual potency) were collected at 1, 3, 6 and 12 month and every 6 months thereafter. Until the end of the study, only one patient was incontinent and 20.7% were impotent. The authors concluded that the age was a predictor of urinary and erectile function recovery in 12 months and the body mass index was significant factor for potency recovery.

With the introduction of robotic surgery, some technical difficulties in laparoscopic surgery were lessened, due to, among other factors, the three-dimensional field of vision, hand tremor filtration and greater ergonomic freedom of movement of the surgeon (2-7). RARP in comparison with the open radical prostatectomy is associated with smaller positive surgical margins for pT2 tumors and better sexual function results during 12 months, and less impairment of urinary function during 12 months (8).

Retrospective studies indicate that urinary control rates are better in younger patients, although there is conflicting data in the literature (9-12). This can be explained by the degeneration of the rhabdosphincter, which occurs with age. In this paper the authors had 16 patients with body mass index (BMI) ≥ 30 kg/m2 at the time of surgery and there was no statistical difference in recovery of urinary continence compared to patients with BMI < 30 kg/m2, but the average time to reach urinary continence was almost double for obese patients. The present paper also confirms that the age in an important factor to impotence recovery. We congratulate the authors for this very important contribution.

Luciano A. Favorito, MD, PhD Professor Associado da Unidade de Pesquisa Urogenital da Universidade do Estado de Rio de Janeiro Urologista do Hospital da Lagoa Federal, Rio de Janeiro Editor Associado da International Braz J Urol
